# Diabetes influences liver stiffness in chronic hepatitis C patients with and without virological cure: A longitudinal study

**DOI:** 10.6061/clinics/2021/e3236

**Published:** 2021-10-28

**Authors:** Daniela Malta Pontual, Leticia Cancella Nabuco, Ronir Raggio Luiz, Ana Carolina Cardoso, Renata M. Perez, Cristiane A. Villela-Nogueira

**Affiliations:** I Programa de Pos-Graduacao em Medicina Interna, Faculdade de Medicina, Universidade Federal do Rio de Janeiro, Rio de Janeiro, RJ, BR.; II Divisao de Hepatologia, Hospital Universitario Clementino Fraga Filho, Universidade Federal do Rio de Janeiro, Rio de Janeiro, RJ, BR.; III Instituto de Estudos de Saude Coletiva, Universidade Federal do Rio de Janeiro, Rio de Janeiro, RJ, BR.; IV Departamento de Gastroenterologia, Universidade do Estado do Rio de Janeiro, Rio de Janeiro, RJ, BR.; V Instituto D’Or de Ensino e Pesquisa, Rio de Janeiro, RJ, BR.; VI Departamento de Medicina Interna, Faculdade de Medicina, Universidade Federal do Rio de Janeiro, Rio de Janeiro, RJ, BR.

**Keywords:** Chronic Hepatitis C, Elastography, Sustained Virological Response, Diabetes Mellitus

## Abstract

**OBJECTIVES:**

The aim was to prospectively assess the variation in liver stiffness (LS) and the associated factors for LS progression in a cohort of naïve, non-responder (NR), and sustained virological response (SVR) chronic hepatitis C (CHC) patients.

**METHODS:**

This was a longitudinal study on CHC patients prospectively followed with serial elastography (Fibroscan®). The LS progression rate was determined, and the associated factors for progression were assessed using multiple linear regression analysis.

**RESULTS:**

A total of 406 patients were followed up for 44 (35-53) months [naïve (29%), NR (24%), and SVR (47%)]. At the end of the follow-up period, the SVR group had a significant decrease in LS [11.8 (9.2) *vs.* 8.8 (8.4) kPa (*p*<0.001)], the NR group had a significant increase in LS [6.6 (5.2) *vs.* 7.1 (4.5) kPa (*p*=0.069)], and the naïve group had no change in LS [6.3 (3.0) *vs.* 6.0 (3.8) kPa (*p*=0.22)]. The related factors for LS progression were lack of SVR (*p*=0.002) and diabetes (*p*=0.05). In the non-diabetic SVR group, a negative rate of progression (-0.047 kPa/month) was observed, whereas in the diabetic SVR group, a positive rate of progression (+0.037 kPa/month) was observed. The highest rate of progression was observed in NR with diabetes at the rate of +0.044 kPa/month.

**CONCLUSION:**

LS in diabetes patients progresses despite SVR, suggesting the need for a close follow-up of this group post-treatment considering the risk of progression of liver disease even after SVR.

## INTRODUCTION

Liver biopsy has recently been replaced by serological or physical non-invasive methods for fibrosis staging in several chronic liver diseases and mainly in chronic hepatitis C (CHC) (1,2). Among the physical non-invasive methods, transient hepatic elastography (THE) is the most widely used point-of-care method; it is painless, easy to perform, and effective for assessing liver fibrosis with well validated cut-off points in CHC (3).

Although THE has been largely validated and considered an accurate tool for the diagnosis of fibrosis staging before HCV treatment (4,5), its role as a follow-up method remains debatable as well as its interpretation and applicability after a sustained virological response (SVR) in CHC patients. Several studies have demonstrated that liver stiffness (LS) assessed using THE decreases in patients with CHC after SVR (6-10). However, although most studies have shown a decrease in LS in SVR patients, LS changes are scarcely evaluated in naïve patients and non-responders (NR) in the direct-acting antiviral (DAA) treatment era, since most studies evaluate only SVR patients. This information might be important mainly in countries where HCV treatment with DAA is not easily available. In addition, the associated factors related to the rate of progression/regression of LS in hepatitis C patients independent of SVR have rarely been evaluated; this evaluation may aid in the follow-up of HCV-infected patients. Therefore, the aim of the present study was to analyze the changes in LS and its associated factors over a long-term follow-up of a large cohort of naïve, NR, and SVR chronic HCV-infected patients.

## PATIENTS AND METHODS

### Study design and patients

Patients with CHC who visited the outpatient liver clinic at the University Hospital of the Federal University of Rio de Janeiro, Brazil, were prospectively included. The patients that compounded the study consisted of three different groups of CHC patients: those who had never received any HCV treatment and remained without treatment throughout the study period (naïve group), those who had failed the previous interferon-based treatment and did not receive any DAA treatment during the study period (NR group), and those who were previously naïve or NR and were treated with DAA during the study period. All patients were included in the study at the same time, irrespective of their group, and treatment was not postponed for any patient. This was possible since the Ministry of Health in Brazil established a priority for treating the sickest patients first at the time the study was conceived. The diagnosis of CHC was based on the presence of anti-HCV antibodies plus a detectable HCV-RNA in serum. Patients with other chronic liver diseases, alcohol ingestion >20g per day, HCV infection and already on DAA treatment, diagnosis of hepatocellular carcinoma, and who underwent any solid organ transplantation such as liver and kidney transplantation were excluded. Patients with cholestasis, ascites, or aminotransferase levels five times higher the upper normal limit were also excluded owing to their effect on THE reliability. Patients who failed the ongoing DAA treatment, developed any complication during treatment (such as hepatocellular carcinoma or need for solid organ transplant), or were lost to follow-up were excluded.

### Ethics

The local ethics committee approved the study (approval number CAE 56934416.000.5257, registered at http://www.plataformabrasil.saude.gov). All patients signed an informed consent form.

### Demographic, Clinical, and Laboratory data

Baseline demographic, anthropometric, clinical, and laboratory data of all patients were registered at the time of the first LS measurement as follows: sex and age (years); body mass index (BMI; kg/m^2^), weight (kg), and abdominal circumference (cm); diagnosis of type 2 diabetes mellitus (DM2) (11) and presence of systemic arterial hypertension (SAH); and (12) aspartate aminotransferase (AST, UI/L), alanine aminotransferase (ALT, UI/L), gamma-glutamyl transferase (GGT, UI/L), platelet count (×10^3^/L), prothrombin time (second), and albumin (g/dL). Data on alcohol consumption and laboratory parameters were registered at baseline and at each THE.

### LS measurement

LS was measured using THE with the FibroScan^®^ Touch 502 equipment (Echosens, Paris, France) as previously described (13). Serial THE of each patient was performed at a minimum interval of 6 months either with the M probe or XL probe. The XL probe was used when LS measurement was unreliable with the M probe. The same probe was used for all measurements for each patient. The fasting interval between the last food intake and THE was at least 3 hours. Ten measurements were obtained; final LS results were expressed in kPa. Unreliable measurements were defined as an interquartile range (IQR) to median value ratio >30% or a success rate (SR) <60% (13). The fibrosis stages based on the obtained LS were defined according to Castéra et al. (2) as follows: LS <7.1 kPa, absence of fibrosis or minimum fibrosis (F0/F1); 7.1-9.4 kPa, moderate fibrosis (F2); 9.5-12.4 kPa, advanced fibrosis (F3); ≥12.5 kPa, cirrhosis (F4). The same cut-offs were adopted for the XL probe. In addition to LS, the controlled attenuation parameter (CAP) evaluated liver steatosis. Both LS and CAP were obtained simultaneously and using the same volume of liver parenchyma. Individuals who underwent treatment had at least one LS measurement at baseline, before treatment, and at least one THE evaluation 6 months post-treatment.

### Statistical analysis

Statistical analysis was performed using SPSS (IBM SPSS Statistics, V.24.0. Armonk, NY). Continuous variables with parametric distribution are expressed as mean±SD, and non-parametric variables are expressed as medians and interquartile ranges. Categorical variables are expressed as absolute numbers and percentages. Continuous variables were compared using ANOVA or the Wilcoxon test. Categorical variables were compared using the chi-square test. Simple linear regression was applied to calculate the rate of progression in each group. To identify variables independently associated with the rate of progression, multiple linear regression analysis was performed. The outcome variable was defined as the rate of progression of LS. A statistical level of 5% was adopted.

## RESULTS

At baseline, 426 chronic HCV patients were enrolled for the evaluation of LS using THE. Twenty patients were excluded after the first LS measurement owing to the following reasons: death because of cirrhosis complications (n=5), liver transplantation (n=4), hepatocellular carcinoma (n=4), additional diagnosis of solid organ cancer (n=3), and lost to follow-up (n=4). Thus, 406 patients completed the study and were followed up for 44 (35-53) months. Patients were categorized according to their status of HCV treatment. Thus, 117 (29%) were classified as naïve from the beginning until the end of the study, 96 (24%) as NR to peg-interferon and ribavirin who did not receive DAA treatment until the end of follow-up, and 193 (47%) as those who fulfilled the criteria for DAA treatment according to the Brazilian Ministry of Health and were included in the study after undergoing the baseline THE. Patients who compounded the last group and achieved SVR comprised the SVR group and underwent at least one LS measurement 6 months post-treatment. The comparative analysis among baseline demographic, anthropometric, clinical, and laboratory characteristics of all patients is shown in Table 1. The naïve group was followed up for 42 (34-51) months, and the median number of THE examinations performed in this group was 3 (2-6). The NR group was followed up for 42 (33-51) months, and the median number of THE examinations performed in this group was 3 (2-6). The SVR group was followed up for 17 (6-99) months after SVR, and the median number of THE examinations performed in this group was 4 (2-6). A comparative analysis of anthropometric data, laboratory data, LS measurement, and CAP was also performed for the three groups at baseline and the last follow-up evaluation (Table 2).

Patients from both the SVR and NR groups showed weight gain with a significant increase in BMI, even though CAP values remained stable over time.

The SVR group showed a significant decrease in LS at baseline and the last follow-up [11.8 (9.2) *vs*. 8.8 (8.4) kPa; *p*<0.01], the NR group showed an increase in LS [6.6 (5.2) *vs*. 7.1 (4.5) kPa; *p*=0.069)], and the naïve group showed no change in LS [6.3 (3.0) *vs*. 6.0 (3.8) kPa; *p*=0.22].

The respective rates of variation based on serial LS evaluation were +0.04, +0.02, and -0.33 kPa/month for the naïve, NR, and SVR groups, respectively.

**Figure 1:**
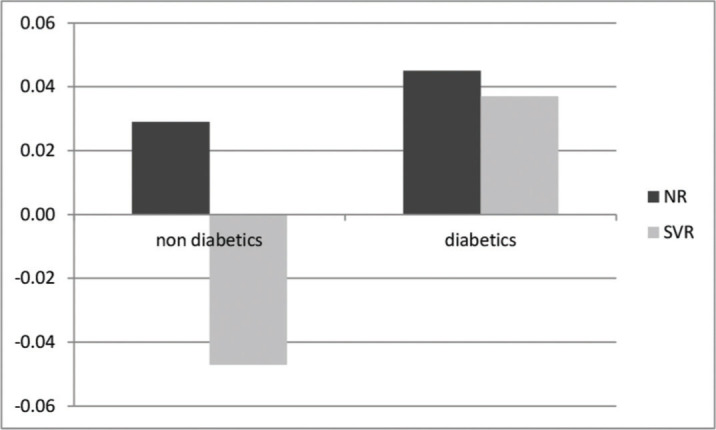
Variation rate of LS measurements with respect to the diagnosis of diabetes mellitus in sustained virological response (SVR) and non-responder (NR) patients.

On multivariate linear regression analysis, achievement of SVR and absence of DM were independently associated with reduction in LS as shown in Table 3. On determination of the rates of variation with respect to the diagnosis of DM and achievement of SVR, the best scenario was observed in the SVR group in patients without DM who showed a negative variation rate of -0.047 kPa/month. The worst scenario was observed in the NR group in diabetes patients who showed the highest progression rate of +0.04 kPa/month. Surprisingly, patients who had achieved SVR but had a diagnosis of DM showed a positive rate of variation of +0.037 kPa/month, while NR without DM showed a positive rate of variation of +0.028 kPa/month (*p*=0.04). The different rates of variation with respect to the diagnosis of DM and achievement of SVR are shown in Figure 1.

## DISCUSSION

This study evaluated changes in LS of a large cohort of HCV-infected patients with different status of treatment: naïve, NR, and SVR. The main finding was that the diagnosis of DM independently affected LS over time. In the present study, SVR patients with DM showed worse progression of LS than NR without diabetes. Patients with CHC have an increased risk of DM and insulin resistance, and the finding that DM may impact fibrosis evolution is worrisome (14). Diabetes is a risk factor for fibrosis progression in non-alcoholic fatty liver disease patients, but its effect in CHC patients has not been prospectively evaluated (15). Fernandes et al. reported a similar effect of diabetes in CHC patients but in a retrospective study including only SVR patients, without the possibility to compare SVR patients with NR or naïve patients (9). The prognostic impact of DM observed in the present study is relevant to clinical practice as it might suggest that patients with DM should remain under care despite virological cure. In our study, BMI had a modest increase in the SVR group, although CAP measurements did not have a significant change over time. It is possible that the impact of diabetes is related to other mechanisms such as persistence of any grade of inflammation, albeit the eradication of HCV. This hypothesis cannot be confirmed owing to the lack of liver biopsy for patients included in this study. In fact, studies including a liver biopsy in the follow-up of HCV-infected patients after DAA treatment are scarce. Martínez-Campreciós et al. recently described a reduction in LS in 8 of 10 patients who underwent a liver biopsy during an elective surgery for hepatocellular carcinoma after SVR (16). They observed that stage 4 fibrosis was still found on liver biopsy even among those who had decreased LS values after SVR. However, they did not evaluate the impact of DM or any metabolic factors related to this outcome, probably owing to the small number of patients who underwent liver biopsy. A study by Pan et al. evaluated 15 patients with paired biopsies before and after SVR via morphometry. They demonstrated that although 11 patients among those who underwent a liver biopsy had F3-F4 fibrosis, there was a 46% reduction in collagen content in 10 patients (17). This suggests that although THE may overestimate cirrhosis’ regression, it is in accordance with changes in the collagen content of the liver leading to a regression of fibrosis during a long-term follow-up period. Thus, the finding of less improvement in LS in patients with SVR should be considered and the factors contributing to this lack of improvement should be evaluated closely. As it is not feasible to indicate liver biopsy for HCV-infected patients before treatment or after SVR in real-world clinical settings, our study provides evidence that patients with a diagnosis of DM should be followed up on carefully since LS (and possibly liver fibrosis) may not improve as expected in the SVR population if diabetes is present. We cannot make any inference regarding the prognosis of LS in the post-treatment period since we did not investigate the outcome of patients included in the present study.

In the present study, LS was assessed in HCV-infected patients with different treatment statuses who were followed for 35-53 months. Many studies have reported a decrease in LS values after SVR, but most included a smaller sample or shorter period of follow-up time (4-13 months) than the present study (6,8,18,19). In additional studies, LS data were available only at the end of treatment, with comparisons of different methods at baseline and the end of follow-up such as THE *vs.* liver biopsy; for example, Cordero-Ruiz et al. described LS changes over 13 years but only in 66 patients and compared distinct methods, which was a possible confounder (20). In addition, our study prospectively followed 193 patients who maintained SVR for more than a year (median of 17 months) after achieving SVR, with a median of four THE examinations for LS evaluation. Furthermore, we also estimated for the first time the ratio of progression/regression of LS using serial THE examinations. Although this ratio was not linear, the rate of progression in the SVR group was negative, -0.33 kPa/month, confirming a decrease in LS in SVR patients; naïve patients and NR had positive rates of +0.04 and +0.02 kPa/month, respectively, showing a trend for the progression of liver fibrosis. The calculation of this ratio is interesting as it is an objective measure to consider the evolution of LS and implement strategies for evaluation of post-treatment patients over time. On calculation of rates considering the diagnosis of diabetes, it is striking how the rate in the SVR group becomes positive, demonstrating that the achievement of SVR in patients with DM may possibly impact the regression of LS and liver fibrosis.

So far, most studies have focused on the follow-up of SVR patients (6,8,9,19,21), describing the linear relationship of SVR and improvement of LS using several non-invasive methods such as THE and acoustic radiation force impulse. Currently, HCV treatment is effective in most DAA-treated patients; however, access to treatment remains an issue in many countries, mainly those underdeveloped. Therefore, a finding that patients who are NR and have diabetes present the worst scenario regarding LS progression is of utmost importance to make treatment available to this population as early as possible.

Few studies have included NR and LS changes in limited samples and some have included patients who were still treated with interferon, varying from 9 to 52 patients overall (6,18,22,23). Our study evaluated 96 NR for a median follow-up period of 42 months. The longest follow-up time recorded so far has been only 20 months, which represents about half the time of our study. However, the results for LS changes are controversial. Studies by Arima et al. and Hézode et al. (6,22) found an improvement in LS, but that by Wang et al. did not confirm this finding (18). In addition, Tada et al. showed a steady LS measurement in NR over time (23). Our results are related to those of patients who were previously treated with pegylated interferon and showed an increase in LS values at the end of the follow-up period. We can confirm this finding by comparing elastography measurements at baseline and the end of follow-up and also by considering the positive ratio of progression obtained for this group (+0.02 kPa/month). We hypothesize that the increase in LS observed in this group was owing to the long follow-up period, which might reinforce the evidence that NR have an increase in LS during a long period of follow-up. Similarly, the long period of follow-up for naïve patients as presented in this study is rarely described in other studies. Erman et al. published a meta-analysis wherein changes in LS were evaluated in 5874 naïve patients with most being coinfected with HIV (24). However, these populations are not comparable with the HCV-monoinfected population owing to faster liver fibrosis progression in HIV-coinfected patients as demonstrated in the referred meta-analysis.

Our study has some limitations owing to the absence of a regular interval between LS evaluations using THE, which prevented analysis of the precise point in time of LS improvement in SVR patients. In addition, we do not have data regarding pre- and post-treatment liver histology owing to its invasiveness and risks. Consequently, we may not be able to state if the improvement in LS was related to the improvement in inflammation or fibrosis and if diabetes influences inflammation through activation of other pathways related to glucose dysmetabolism. Despite these limitations, we did study three different groups of patients (NR, naïve, and SVR) including 194 SVR patients followed up for 17 months (70 weeks). Compared with the multicenter German hepatitis C-registry study published recently (19) that evaluated 260 patients over 6 months, our unicenter, longitudinal real-world study presents a long-term follow-up of 17 months of Brazilian hepatitis C patients.

## CONCLUSION

SVR patients showed a significant improvement in LS over a long-term follow-up period, when compared with NR and naïve HCV-infected patients. However, even in patients with SVR, DM influences the improvement in LS over time. Therefore, DM may be considered a marker of worse prognosis in HCV-infected patients. Follow-up of diabetes patients should not be discontinued even after they achieve SVR. In addition to patients with advanced fibrosis, patients with DM should be prioritized when defining HCV treatment strategies in low-income countries.

## AUTHOR CONTRIBUTIONS

Pontual DM collected the data, followed up on the patients, and reviewed the manuscript. Nabuco LC interpreted the data and reviewed the manuscript. Luiz RR performed statistical analysis, interpreted the data, and reviewed the manuscript. Cardoso AC collected the data and reviewed the manuscript. Perez RM interpreted the data and reviewed the manuscript. Villela-Nogueira CA conceived the study, analyzed the data and interpreted the results, and drafted and reviewed the final version of the manuscript.

## Figures and Tables

**Table 1:** Baseline characteristics and comparative analysis of the HCV-infected naïve, NR, and SVR groups (n=406).

Variable	Total (n=406)	Naïve (n=117)	NR (n=96)	SVR (n=193)	*p*-value
Age, years	58.9±0.7	58.2±1.4	59.4±1.2	59.1±1.0	0.14
Female, (%)	246 (61)	73 (30)	55 (22)	118 (48)	0.59
Weight, kg	70.3±0.9	68.1±1.5	70.3±1.9	71.9±1.3	0.71
BMI, kg/m^2^	26.3±0.3	25.8±0.5	26.2±0.6	26.7±0.4	0.76
DM, (%)	87 (21)	25 (29)	24 (28)	38 (44)	0.58
SAH, (%)	195 (48)	50 (26)	52 (27)	92 (47)	0.27
CKD, (%)	14 (3)	5 (36)	4 (29)	5 (36)	0.66
ALT (IU/L)	66 (52)	60 (45)	54 (28)	76 (67)	0.001
AST (IU/L)	48 (37)	40 (30)	40 (27)	56 (55)	<0.01
GGT (IU/L)	67 (90)	59 (77)	54 (59)	88 (99)	0.01
Albumin (g/dL)	4.0 (0.6)	4.0 (0.5)	4.0 (0.7)	4.0 (0.6)	0.90
Platelets (×10^3^)	175±67	195±63	165±64	177±73	0.001*
Elastography, kPa	8.4(5.9-13.6)	6.1 (5.1-7.7)	6.6 (5.2-10.4)	11.8 (8.5-17.2)	0.001**
CAP (dB/m)	229±48	216±54	235±37	237±50	0.04***
Follow-up time (months)	44 (35-53)	42 (34-51)	42 (33-51)	17 (6-99)	
HCV Genotypes§	n (%)				0.83
1b	173 (44)	54 (48)	35 (37)	84 (44)	
1a	139 (35)	34 (30)	39 (42)	66 (35)	
1	52 (13)	14 (12)	13 (14)	26 (14)	
2	02 (0.5)	01 (0.5)	0 (0)	01 (0.5)	
3	30 (7.5)	9 (8)	7 (7.5)	14 (7)	
METAVIR Fibrosis staging according to LS^5^	n (%)				0.01
F0/F1	160 (39)	79 (68)	50 (52)	31 (16)	
F2	70 (17)	21 (18)	17 (18)	32 (17)	
F3	67 (17)	7 (6)	13 (13)	47 (24)	
F4	109 (27)	10 (8)	16 (17)	83 (43)	

Values are presented as mean and standard deviation or median and interquartile range. BMI: body mass index; DM: diabetes mellitus; SAH: systemic arterial hypertension; HCV, Hepatitis C virus; HIV: human immunodeficiency virus; CKD: chronic kidney disease; ALT: alanine aminotransferase; AST: aspartate aminotransferase; GGT: gamma-glutamyl transferase; LS: liver stiffness; CAP: controlled attenuation parameter; NR: previous non-responders to PEG-IFN; SVR: sustained virological response.

* Statistical significance between the naïve and SVR groups, *p*-value 0.001;

** Statistical significance between the naïve and SVR and NR and SVR groups, *p*-value 0.001;

*** Statistical significance between the naïve and NR groups, *p*-value 0.014.

§ Genotype was unknown in 12 patients.

**Table 2:** Comparative analysis of the naïve, NR, and SVR HCV-infected patients at baseline and at end of follow-up (n=406).

	Naïve (n=117)			NR (n=96)			SVR (n=193)		
Variables	Baseline	End of follow-up 47 (38-56) Months	*p*	Baseline	End of follow-up 42 (33-51) months	*p*	Baseline	End of follow-up 17 (6-99) months	*p*
Weight (kg)	68.7±12.8	68.8±13.3	0.056	72.2±13.6	74.4±13.7	0.004	72.2±13.3	74.0±14.3	<0.001
BMI (kg/m^2^)	25.9±4.2	26.0±4.4	0.051	26.9±4.3	27.6±3.9	0.012	27.1±4.5	27.7±4.7	<0.001
ALT (IU/L)	58 (44-83)	56 (38-84)	0.10	61 (47-84)	56 (42-79)	0.06	75 (45-112)	24 (18-38)	<0.001
AST (IU/L)	40 (30-60)	43 (27-60)	0.95	45 (31-57)	47 (33-64)	0.81	56 (34-87)	26 (21-31)	<0.001
GGT (IU/L)	60 (36-102)	55 (31-99)	0.54	59 (35-108)	68 (35-122)	0.80	85 (51-142)	30 (22-60)	<0.001
Elastography (kPa)	6.1 (5.1-7.7)	6.1 (4.9-7.9)	0.22	6.6 (5.2-10.4)	7.1 (5.6-10.1)	0.069	11.8 (8.5-17.2)	8.8 (6.0-14.4)	<0.001
CAP (dB/m)	217±55	220±56	0.68	237±39	234±46	0.26	231±47	236±56	0.39

Values are presented as mean and standard deviation or median and interquartile range. NR: previous non-responders; SVR: sustained virological response; BMI: body mass index; ALT: alanine aminotransferase; AST: aspartate aminotransferase; GGT: gamma-glutamyl transferase; CAP: controlled attenuation parameter.

**Table 3:** Independent factors related to the rate of progression of LS in HCV-infected patients (n=406).

Variables	Beta- Coefficient	95% CI	*p*-value
Diabetes Mellitus	0.047	(0.001; 0.094)	0.05
SVR	-0.062	(-0.101; -0.023)	0.002

LS, liver stiffness; HCV, Hepatitis C virus; SVR, sustained virological response; CI, confidence interval.
